# A Cross-Study Biomarker Signature of Human Bronchial Epithelial Cells Infected with Respiratory Syncytial Virus

**DOI:** 10.1155/2016/3605302

**Published:** 2016-05-04

**Authors:** Luiz Gustavo Gardinassi

**Affiliations:** Department of Biochemistry and Immunology, Ribeirão Preto Medical School, University of São Paulo, 14049-900 Ribeirão Preto, SP, Brazil

## Abstract

Respiratory syncytial virus (RSV) is a major cause of lower respiratory tract infections in children, elderly, and immunocompromised individuals. Despite of advances in diagnosis and treatment, biomarkers of RSV infection are still unclear. To understand the host response and propose signatures of RSV infection, previous studies evaluated the transcriptional profile of the human bronchial epithelial cell line—BEAS-2B—infected with different strains of this virus. However, the evolution of statistical methods and functional analysis together with the large amount of expression data provide opportunities to uncover novel biomarkers of inflammation and infections. In view of those facts publicly available microarray datasets from RSV-infected BEAS-2B cells were analyzed with linear model-based statistics and the platform for functional analysis InnateDB. The results from those analyses argue for the reevaluation of previously reported transcription patterns and biological pathways in BEAS-2B cell lines infected with RSV. Importantly, this study revealed a biosignature constituted by genes such as* ABCC4*,* ARMC8*,* BCLAF1*,* EZH1*,* FAM118A*,* FAM208B*,* FUS*,* HSPH1*,* KAZN*,* MAP3K2*,* N6AMT1*,* PRMT2*,* S100PBP*,* SERPINA1*,* TLK2*,* ZNF322*, and* ZNF337* which should be considered in the development of new molecular diagnosis tools.

## 1. Introduction

Respiratory syncytial virus (RSV) is a major etiologic agent causing acute lower respiratory infections that can progress to bronchiolitis and pneumonia in children, elderly, and immunocompromised individuals [1, 2]. RSV outbreaks are influenced by virus diversity and evolution [3, 4], environmental factors [5], and host immunity [6].

The epithelium is the primary site for host-virus interface, where cells recognize pathogen-associated patterns on microbes through innate immunity receptors [7, 8]. Indeed, epithelial cells constitute an important line of defense against RSV and other airborne pathogens [9]. They form a physical barrier and produce mucus to inhibit microbes from entering the body. Moreover, they express molecules with antimicrobial properties, as lysozyme, lactoferrin, collectins, and antimicrobial peptides [10]. Two human cell lines have been extensively used to understand the interaction between host and RSV, the alveolar epithelial cell, A549, and one from proximal airways, the bronchial epithelial cell, BEAS-2B.

Genome-wide microarrays are powerful tools to investigate host transcriptional response during infections in the pulmonary epithelium, including those induced by RSV [11, 12]. Indeed, two studies evaluated the patterns of gene expression from BEAS-2B cell lines infected with RSV [10, 13]. However, it is intriguing that after 4 h of infection Huang and collaborators (2008) found that RSV-modulated genes were only associated with the neuroactive ligand-receptor interaction pathway [13]; in contrast, Mayer and collaborators (2007) identified that the same time of RSV infection of BEAS-2B cells induced transcriptional changes similar to those found for other respiratory pathogens as* Pseudomonas aeruginosa* [10]. In spite of differences, publicly available microarray data offers an interesting opportunity to reveal common features of RSV induced transcriptional profiles to understand the early response of BEAS-2B cell lines and extend the knowledge on biomarkers of acute infections with this virus. Therefore, those datasets were evaluated in a meta-analysis by fitting linear models for each array probe and Empirical Bayesian approach to detect transcriptional changes that revealed significant associations with unreported pathways. Of importance, this strategy also rendered a biomarker signature of BEAS-2B cell lines infected with RSV that can be useful for the design of molecular diagnosis tools.

## 2. Materials and Methods

The datasets GSE3397 and GSE6802 were obtained from GEO database (http://www.ncbi.nlm.nih.gov/), which compared BEAS-2B cells infected with RSV with control experiments. Only arrays in which cells were infected with RSV for 4 h were selected for further analysis. Raw data were processed using the R Language and Environment for Statistical Computing (R) 3.2.0 [14] and Bioconductor 3.1 [15]. The* affy* package for R [16] was used to perform quality control when applicable. Data was log_2_⁡  transformed and quantile normalization was applied for dataset GSE3397 due the absence of CEL files. The dataset GSE6802 was already RMA normalized. Batch effects were corrected with Combat( ) function [17] of* sva* package for R [18]. Expression data were weighted with the arrayWeights( ) function from* limma* package for R [19]. Differential gene expression was also evaluated with* limma* package for R [19], whereby differentially expressed genes (DEGs) were identified by a false discovery rate (FDR) <0.05. Hierarchical clustering was performed with Euclidian distance for metric calculations and the complete linkage method, which were displayed as heatmaps drawn with* gplots* package for R [20]. Pathway analyses were performed with the online platform for functional analysis InnateDB [21] and significant pathway overrepresentation was computed with hypergeometrical distribution and Benjamini-Hochberg correction for multiple comparisons. Significantly enriched pathways were determined by a *P* value < 0.05 and FDR < 0.1.

## 3. Results and Discussion

### 3.1. Dataset Selection and Preprocessing Analysis

**Figure 1:**
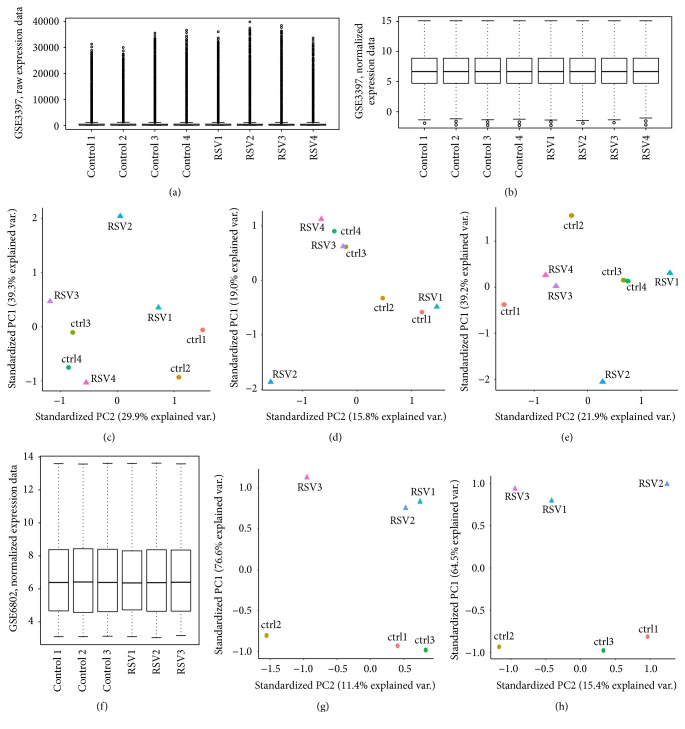
Preprocessing analysis of GEO datasets GSE3397 and GSE6802. (a) Boxplot of GSE3397, raw expression data. (b) Boxplot of GSE3397, normalized expression data. (c) Principal component analysis of GSE3397, raw expression data. (d) Principal component analysis of GSE3397, normalized expression data. (e) Principal component analysis of GSE3397, normalized and batch corrected expression data. (f) Boxplot of GSE6802, RMA normalized expression data. (g) Principal component analysis of GSE6802, normalized expression data. (h) Principal component analysis of GSE6802, log_2_ transformed RMA normalized expression data.

To define a robust transcriptional signature of BEAS-2B acutely infected with RSV, two publicly available datasets, GSE3397 and GSE6802, were used to conduct a meta-analysis from which data were extracted for BEAS-2B cells infected with RSV for 4 h and controls. First, background subtracted expression data from GSE3397 (Figure 1(a)) were preprocessed and normalized (Figure 1(b)). However, in a first attempt to conduct differential gene expression analysis using* limma* [19], there were no statistically significant differences in gene expression. Therefore, principal component analysis (PCA) was used to evaluate the expression profiles of each array and, except for arrays named here Control2 and RSV2, the consistent pattern of clustering in Figure 1(c) suggests a batch effect. After normalization, this effect was even more evident (Figure 1(d)), which led to the speculation that Huang and collaborators (2008) [13] analyzed only three microarray experiments from this dataset based on the assumption that differences found for those microarrays were due to failures in experimental procedures; however they did not consider or correct for batch effects. In view of those facts, the datasets were adjusted with Combat function for R, which removed such effects from GSE3397 expression data (Figure 1(e)). Batch correction of GSE3397 did not change the profiles of arrays Control2 and RSV2; nevertheless, those arrays were included in further analysis because the variation observed in this experiment could have a substantial impact over the final result. Even adverse experimental variations that may change the overall expression patterns of a dataset could be useful to power up the identification of genes that are robustly modulated in BEAS-2B cells infected with RSV. The expression dataset GSE6802 (Figure 1(f)) was also included in the analysis. PCA from expression data extracted from GEO demonstrates that most of the variability between the arrays is explained (76.6%) by the infection with RSV, as the standardized PC1 separates RSV-infected from control arrays (Figure 1(g)), whereas standardized PC2 (11.4%) separates one pair of arrays (RSV_3 and ctrl2) and, although these arrays are supposedly from different batches, clustering features of this axis also suggested a batch effect (Figure 1(g)). log_2_ transformation of data impacted the profile of array RSV_1 however did not change the profiles from RSV_3 and ctrl_2 (Figure 1(h)). Combat( ) function was also applied to the expression dataset GSE6802; however, PCA shows that the adjustment did not to improve further clustering between specific arrays (Supplementary Figure  1; see Supplementary Material available online at http://dx.doi.org/10.1155/2016/3605302). In view of that, downstream analyses were carried out with normalized log_2_ transformed data.

### 3.2. Differential Gene Expression

**Figure 2:**
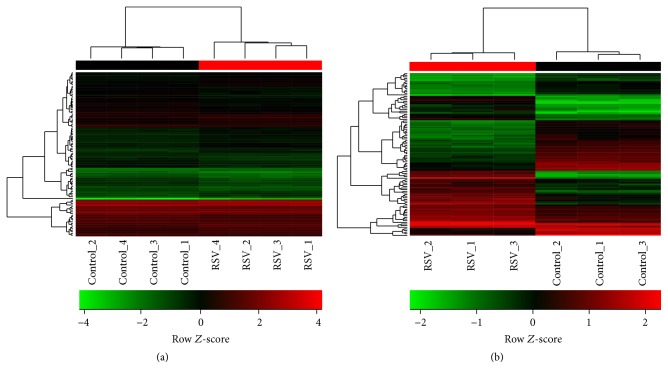
Transcriptional profiles of BEAS-2B cells infected with RSV for 4 h. (a) Hierarchical clustering of differentially expressed genes from dataset GSE3397. (b) Hierarchical clustering of differentially expressed genes from dataset GSE6802. Row *Z*-scores were calculated based on normalized expression data. The colors from green to red represent the transition of decreased to increased expression.

Next, linear model-based statistical analyses with a FDR < 0.05 were conducted to identify differentially expressed genes (DEGs). The dataset GSE3397 exhibited ninety-four DEGs (Figure 2(a) and Table 1). Those genes are highly discordant from DEGs previously reported by Huang and collaborators (2008) [13], which identified 277 DEGs based on different statistical analysis and assumptions. Fifty genes were downregulated and forty-four were upregulated (Table 1). The differences found in this study might reflect the inclusion of all microarray experiments from controls and 4 h after RSV infection; exclusion of expression data from 24 h after RSV infection; distinct preprocessing approaches as normalizing method and batch effect correction; and the assessment of statistical significance with a linear model-based method and corrected *P* values. In contrast, 1965 DEGs were identified for the dataset GSE6802. The top hundred DEGs ranked by fold changes (Figure 2(b) and Table 2) included genes such as* JUNB*,* KLF4*,* CXCL1*,* CXCL2*, and* IL6*, which are in agreement with those reported by Mayer and collaborators (2007) [10]. Several factors should account for the notable differences in expression analysis from both datasets. First, different RSV strains were used to stimulate BEAS-2B cells. Second, experimental conditions of controls were also different, as control experiments from GSE3397 were incubated with vehicle (not specified) and those from GSE6802 were not stimulated. Third, despite both datasets being generated with affymetrix microarray platform, those include distinct versions, HU133 plus 2.0 for GSE3397 and HU133A 2.0 for GSE6802.

### 3.3. Functional Analysis

**Figure 3:**
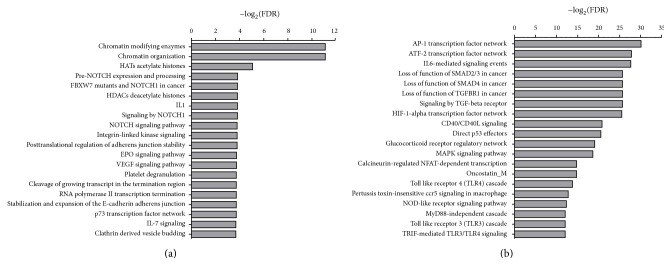
Pathway enrichment analysis with InnateDB. Differentially expressed genes from (a) GSE3397 or (b) GSE6802 were evaluated for overrepresentation in pathways annotated in databases as INOH, KEGG, NETPATH, PID NIC, and REACTOME.

To obtain a biological interpretation of the transcriptional signature of RSV-infected BEAS-2B cells and compare with those reported by previous studies, enrichment analysis was performed with the online platform for functional analysis InnateDB [21]. Based on a FDR < 0.1, DEGs identified for GSE3397 were enriched in pathways related to Chromatin organization, histone acetylation, signaling by NOTCH, IL1, Integrin-linked kinase signaling, EPO signaling pathway, VEGF signaling pathway, platelet degranulation, p73 transcription factor network, IL-7 signaling, p53 signaling pathway, and others (Figure 3(a) and Supplementary Data 1). Of interest, Huang and collaborators (2008) [13] reported gene overrepresentation within p53 signaling pathway, but only after 24 h following RSV infection of BEAS-2B cells. After 4 h following RSV infection, Huang and collaborators (2008) [13] only found a significant association with neuroactive ligand-receptor interaction pathway, which was not overrepresented in the present analysis. In contrast, DEGs resultant from dataset GSE6802 were enriched in pathways related to AP-1 transcription factor, ATF-2 transcription factor, IL-6 signaling, SMAD function, signaling by TGFBR, HIF-1*α* transcription factor, signaling by CD40/CD40L, signaling by MAPK, signaling by innate immune receptors, and others (Figure 3(b) and Supplementary Data 1). Some of those pathways as CD40 signaling are indeed commonly induced by a variety of viral respiratory infections [22], whereas several of those pathways could indicate novel directions for studying the host response against RSV. Six pathways were enriched by DEGs from both datasets, the EPO signaling pathway, FBXW7 Mutants and NOTCH1 in Cancer, IL1, p53 signaling pathway, p73 transcription factor network, and signaling by NOTCH1. The erythropoietin (EPO) gene is a primary target of HIF-1*α* transcription factor, whereas binding of HIF-1*α* to the EPO enhancer promoter region induces transcriptional programs that influence inflammation and infection processes [23]. In addition, expression of Dll4, a major NOTCH ligand, is upregulated in dendritic cells infected with RSV, whereas blockage of Dll4* in vivo* increased hyperreactivity of airways and mucus secretion that impacted the pathology of the disease, showing a key role of signaling by NOTCH in the regulation of immunity against RSV [24]. Moreover, besides modulations of the p53 signaling pathway by infection of RSV* in vitro* [10, 13], this pathway was found to be upregulated in whole blood of children with lower respiratory tract infection by RSV [25]. Taken together, those data point to key pathways which can impact infections of human bronchial epithelial cells with RSV.

### 3.4. Meta-Analysis Based Biomarker Signature of RSV-Infected BEAS-2B Cells

**Figure 4:**
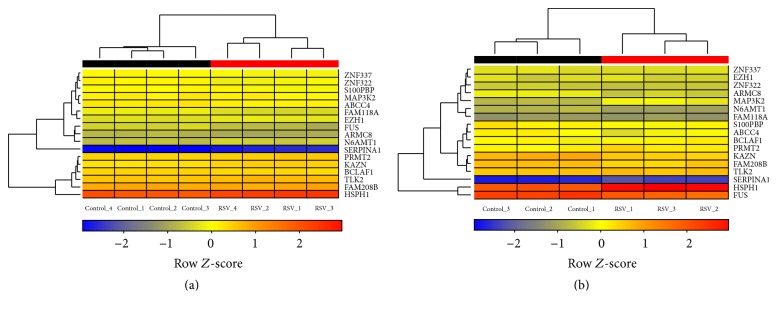
Biomarker signature of BEAS-2B cells infected with RSV for 4 h. Hierarchical clustering of expression data for* ABCC4*,* ARMC8*,* BCLAF1*,* EZH1*,* FAM118A*,* FAM208B*,* FUS*,* HSPH1*,* KAZN*,* MAP3K2*,* N6AMT1*,* PRMT2*,* S100PBP*,* SERPINA1*,* TLK2*,* ZNF322*, and* ZNF337* from (a) dataset GSE3397 and (b) dataset GSE6802. Row *Z*-scores were calculated based on normalized expression data. The colors from blue to red represent the transition of decreased to increased expression.

To determine a unique transcriptional signature of BEAS-2B cells induced by early infection with RSV, common DEGs for both datasets were further identified. The analysis retrieved a list of seventeen common genes:* ABCC4*,* ARMC8*,* BCLAF1*,* EZH1*,* FAM118A*,* FAM208B*,* FUS*,* HSPH1*,* KAZN*,* MAP3K2*,* N6AMT1*,* PRMT2*,* S100PBP*,* SERPINA1*,* TLK2*,* ZNF322*, and* ZNF337* (Figure 4). Despite particular features in expression data from both datasets, unsupervised hierarchical clustering analysis based on this signature revealed the formation of robust clusters between RSV-infected or uninfected BEAS-2B cells (Figure 4). Of note, human airway epithelial cells were shown to express ABCC4/MRP4, a transporter for uric acid and cAMP [26]. Mucosal production of uric acid was recently linked to particulate matter-induced allergic sensitization [26]; therefore RSV infection could trigger such a response and contribute to the development and severity of allergic responses to particulate matter [27]. Moreover, both ABCC4 and SERPINA1 are annotated into the platelet degranulation pathway (Figure 3(a)), suggesting a role in antiviral mechanisms from bronchial epithelial cells. After an initial encounter with RSV, the transcriptional activity of human bronchial epithelial cells is reprogrammed to counteract viruses and other pathogens [10], whereas* MAP3K2* and* ZNF322* are clearly involved on the activation and regulation of MAP kinase signaling pathway [28, 29]. Indeed, RSV infection leads to the activation of p38 MAPK [30] and c-JUN kinase pathway, which negatively regulates the production of TNF-*α* in human epithelial cells [31] and might contribute to virus evasion from an early immune response. Interestingly, the biosignature also included BCLAF1, a molecule involved in processes as apoptosis, transcription and processing of RNA, and export of mRNA from the nucleus [32]. However, this nuclear protein was also implicated as a viral restriction factor targeted to degradation by human cytomegalovirus [32]. Moreover, EZH1 was shown to be involved in the methylation of histone 3 at lysine 27 (H3K27) of the HIV provirus in resting cells [33] and could thus exert a significant function in infections with RSV, whereby other genes such as N6AMT1, FUS, and PRMT2 are also involved in protein methylation. Indeed, using coimmunoprecipitation and mass spectrometry, recent work demonstrated that RSV nucleoprotein (N) interacts with protein arginine N-methyltransferase 5 (PRMT5) [34], suggesting that PRMT2 could also interact with RSV proteins and play an important role during infections of human bronchial epithelial cells. Several of the genes identified in this study have been poorly studied in the context of RSV infection, whereby none of them was previously reported as a biomarker of infections by this virus. Of note, except for* FAM208B* and* KAZN*, analysis conducted by Smith and collaborators (2012) [22] which included both datasets (GSE3397 and GSE6802) also identified the significant modulation of the genes included in the biomarker signature identified herein.

## 4. Conclusions

The combined analysis of distinct datasets from BEAS-2B cells infected with RSV retrieved intriguing results, whereby using powerful statistical methods and assumptions this study identified a new set of biomarkers of early infection with RSV composed by seventeen genes:* ABCC4*,* ARMC8*,* BCLAF1*,* EZH1*,* FAM118A*,* FAM208B*,* FUS*,* HSPH1*,* KAZN*,* MAP3K2*,* N6AMT1*,* PRMT2*,* S100PBP*,* SERPINA1*,* TLK2*,* ZNF322*, and* ZNF337*. This transcriptional signature could be useful for the development of molecular diagnosis tools as well as future investigations of processes involved in host-pathogen interactions.

## Supplementary Material

Pathway enrichment analysis with the web-based platform InnateDB.

## Figures and Tables

**Table 1:** Differentially expressed genes identified in dataset GSE3397.

ProbeID	Gene symbol	Gene name	log_2_ fold change	FDR
1560754_at	CMTM7	CKLF like MARVEL transmembrane domain containing 7	−1,54756	0,017104
239439_at	AFF4	AF4/FMR2 family member 4	−1,53581	0,023832
238929_at	SRSF8	Serine/arginine-rich splicing factor 8	−1,51887	0,018433
223142_s_at	UCK1	Uridine-cytidine kinase 1	−1,47939	0,017104
242636_at	PRCP	Prolylcarboxypeptidase	−1,45095	0,034358
228007_at	CEP85L	Centrosomal protein 85 kDa-like	−1,4103	0,017104
235573_at	HSPH1	Heat shock protein family H (Hsp110) member 1	−1,39959	0,0371
228391_at	CYP4V2	Cytochrome P450 family 4 subfamily V member 2	−1,38799	0,01671
219376_at	ZNF322	Zinc finger protein 322	−1,3491	0,046761
1553689_s_at	METTL6	Methyltransferase like 6	−1,34723	0,017104
242837_at	SRSF4	Serine/arginine-rich splicing factor 4	−1,34071	0,044693
237215_s_at	TFRC	Transferrin receptor	−1,32685	0,017104
208819_at	RAB8A	RAB8A, member RAS oncogene family	−1,32593	0,042264
236665_at	CCDC18	Coiled-coil domain containing 18	−1,31494	0,034201
206147_x_at	SCML2	Sex comb on midleg-like 2 (Drosophila)	−1,30586	0,016454
229325_at	ZZZ3	Zinc finger ZZ-type containing 3	−1,30495	0,017104
1565716_at	FUS	FUS RNA binding protein	−1,29415	0,049505
205062_x_at	ARID4A	AT-rich interaction domain 4A	−1,28877	0,033039
1552312_a_at	MFAP3	Microfibrillar associated protein 3	−1,28521	0,046511
223223_at	ARV1	ARV1 homolog, fatty acid homeostasis modulator	−1,27987	0,023832
232001_at	PRKCQ-AS1	PRKCQ antisense RNA 1	−1,27987	0,035983
233195_at	DNAI1	Dynein axonemal intermediate chain 1	−1,25963	0,047083
219094_at	ARMC8	Armadillo repeat containing 8	−1,25527	0,043392
235232_at	GMEB1	Glucocorticoid modulatory element binding protein 1	−1,2492	0,046511
218643_s_at	CRIPT	CXXC repeat containing interactor of PDZ3 domain	−1,24229	0,0371
1566851_at	TRIM42	Tripartite motif containing 42	−1,24057	0,042149
221821_s_at	KANSL2	KAT8 regulatory NSL complex subunit 2	−1,23799	0,017104
244115_at	FAM126A	Family with sequence similarity 126 member A	−1,23114	0,033039
215541_s_at	DIAPH1	Diaphanous related formin 1	−1,22774	0,033039
203196_at	ABCC4	ATP binding cassette subfamily C member 4	−1,22519	0,033039
225024_at	RPRD1B	Regulation of nuclear pre-mRNA domain containing 1B	−1,22264	0,043765
37860_at	ZNF337	Zinc finger protein 337	−1,22095	0,023832
212997_s_at	TLK2	Tousled like kinase 2	−1,21841	0,04814
225690_at	CDK12	Cyclin-dependent kinase 12	−1,21083	0,0371
232103_at	BPNT1	3′(2′), 5′-Bisphosphate nucleotidase 1	−1,20748	0,0371
224848_at	CDK6	Cyclin-dependent kinase 6	−1,20247	0,0371
214962_s_at	NUP160	Nucleoporin 160 kDa	−1,20247	0,046319
219629_at	FAM118A	Family with sequence similarity 118 member A	−1,19831	0,028374
212290_at	SLC7A1	Solute carrier family 7 member 1	−1,19748	0,042264
227187_at	CBLL1	Cbl proto-oncogene like 1, E3 ubiquitin protein ligase	−1,19582	0,030047
233208_x_at	CPSF2	Cleavage and polyadenylation specific factor 2	−1,19334	0,046319
230566_at	MORC2-AS1	MORC2 antisense RNA 1	−1,17691	0,0371
238795_at	FAM208B	Family with sequence similarity 208 member B	−1,17609	0,0371
204980_at	CLOCK	Clock circadian regulator	−1,17283	0,0371
238653_at	LRIG2	Leucine-rich repeats and immunoglobulin like domains 2	−1,17202	0,048527
229939_at	ENDOV	Endonuclease V	−1,16878	0,041349
218185_s_at	ARMC1	Armadillo repeat containing 1	−1,16151	0,046319
201083_s_at	BCLAF1	BCL2 associated transcription factor 1	−1,15509	0,049505
227840_at	C2orf76	Chromosome 2 open reading frame 76	−1,15109	0,042264
201686_x_at	API5	Apoptosis inhibitor 5	−1,14076	0,046761
221699_s_at	DDX50	DEAD-box helicase 50	1,140764	0,046511
1556178_x_at	TAF8	TATA-box binding protein associated factor 8	1,159096	0,034358
205623_at	ALDH3A1	Aldehyde dehydrogenase 3 family member A1	1,163927	0,049505
212495_at	KDM4B	Lysine demethylase 4B	1,193336	0,044693
1569057_s_at	MIA3	Melanoma inhibitory activity family member 3	1,193336	0,047866
222494_at	FOXN3	Forkhead box N3	1,19582	0,048527
223311_s_at	MTA3	Metastasis associated 1 family member 3	1,19582	0,041439
215424_s_at	SNW1	SNW domain containing 1	1,196649	0,049505
213478_at	KAZN	Kazrin, periplakin interacting protein	1,19914	0,025143
227864_s_at	MVB12A	Multivesicular body subunit 12A	1,201636	0,030287
228674_s_at	EML4	Echinoderm microtubule associated protein like 4	1,204137	0,040345
224196_x_at	DPH5	Diphthamide biosynthesis 5	1,205808	0,025143
224652_at	CCNY	Cyclin Y	1,207481	0,046761
212968_at	RFNG	RFNG O-fucosylpeptide 3-beta-N-acetylglucosaminyltransferase	1,211673	0,0371
1555486_a_at	PRR5L	Proline rich 5 like	1,212513	0,017104
232837_at	KIF13A	Kinesin family member 13A	1,214195	0,042264
224320_s_at	MCM8	Minichromosome maintenance 8 homologous recombination repair factor	1,217566	0,033039
230131_x_at	ARSD	Arylsulfatase D	1,221793	0,0371
218225_at	ECSIT	ECSIT signalling integrator	1,224336	0,034358
222610_s_at	S100PBP	S100P binding protein	1,226885	0,030047
32259_at	EZH1	Enhancer of zeste 1 polycomb repressive complex 2 subunit	1,229439	0,0371
203854_at	CFI	Complement factor I	1,232852	0,042264
221600_s_at	AAMDC	Adipogenesis associated, Mth938 domain containing	1,260503	0,0371
209558_s_at	HIP1R	Huntingtin interacting protein 1 related	1,263127	0,042264
224814_at	DPP7	Dipeptidyl peptidase 7	1,26488	0,016454
232280_at	SLC25A29	Solute carrier family 25 member 29	1,277214	0,030047
228424_at	NAALADL1	N-Acetylated alpha-linked acidic dipeptidase-like 1	1,286989	0,042264
203409_at	DDB2	Damage specific DNA binding protein 2	1,288775	0,023832
229975_at	BMPR1B	Bone morphogenetic protein receptor type 1B	1,297739	0,034358
227073_at	MAP3K2	Mitogen-activated protein kinase kinase kinase 2	1,297739	0,017104
225347_at	ARL8A	ADP ribosylation factor like GTPase 8A	1,298639	0,02672
221774_x_at	SUPT20H	SPT20 homolog, SAGA complex component	1,308578	0,016454
223679_at	CTNNB1	Catenin beta 1	1,318594	0,018043
227679_at	HDAC11	Histone deacetylase 11	1,328686	0,044693
220020_at	XPNPEP3	X-Prolyl aminopeptidase 3, mitochondrial	1,342573	0,031097
203199_s_at	MTRR	5-Methyltetrahydrofolate-homocysteine methyltransferase reductase	1,360371	0,017104
228722_at	PRMT2	Protein arginine methyltransferase 2	1,370783	0,016454
228951_at	SLC38A7	Solute carrier family 38 member 7	1,431969	0,016454
217529_at	ORAI2	ORAI calcium release-activated calcium modulator 2	1,453973	0,043775
220311_at	N6AMT1	N-6 adenine-specific DNA methyltransferase 1 (putative)	1,460032	0,017104
213402_at	ZNF787	Zinc finger protein 787	1,469169	0,017104
226055_at	ARRDC2	Arrestin domain containing 2	1,477338	0,017104
219756_s_at	POF1B	Premature ovarian failure, 1B	1,580083	0,016454
202833_s_at	SERPINA1	Serpin peptidase inhibitor, clade A (alpha-1 antiproteinase, antitrypsin), and member 1	2,488023	0,0371

**Table 2:** Top hundred differentially expressed genes identified in dataset GSE6802.

ProbeID	Gene symbol	Gene name	log_2_ fold change	FDR
212615_at	CHD9	Chromodomain helicase DNA binding protein 9	−3,69609	0,00131
221840_at	PTPRE	Protein tyrosine phosphatase, receptor type E	−3,56524	0,000195
220817_at	TRPC4	Transient receptor potential cation channel subfamily C member 4	−3,39168	0,001582
221703_at	BRIP1	BRCA1 interacting protein C-terminal helicase 1	−2,88786	0,021463
207012_at	MMP16	Matrix metallopeptidase 16	−2,82647	0,000119
219494_at	RAD54B	RAD54 homolog B (S. cerevisiae)	−2,81279	0,000177
207034_s_at	GLI2	GLI family zinc finger 2	−2,79723	0,005157
203518_at	LYST	Lysosomal trafficking regulator	−2,75872	5,90*E* − 05
205282_at	LRP8	LDL receptor related protein 8	−2,7549	0,000311
214440_at	NAT1	N-Acetyltransferase 1 (arylamine N-acetyltransferase)	−2,68515	0,001777
219627_at	ZNF767P	Zinc finger family member 767, pseudogene	−2,67957	0,00024
218984_at	PUS7	Pseudouridylate synthase 7 (putative)	−2,67586	0,001308
206554_x_at	SETMAR	SET domain and mariner transposase fusion gene	−2,63536	0,002432
219779_at	ZFHX4	Zinc finger homeobox 4	−2,62624	0,001411
213103_at	STARD13	StAR related lipid transfer domain containing 13	−2,57219	0,002525
210138_at	RGS20	Regulator of G-protein signaling 20	−2,55974	0,000415
204291_at	ZNF518A	Zinc finger protein 518A	−2,54383	9,70*E* − 05
204651_at	NRF1	Nuclear respiratory factor 1	−2,49147	0,003659
205408_at	MLLT10	Myeloid/lymphoid or mixed-lineage leukemia; translocated to, 10	−2,48975	5,10*E* − 05
219581_at	TSEN2	tRNA splicing endonuclease subunit 2	−2,45377	0,001774
218242_s_at	SUV420H1	Lysine methyltransferase 5B	−2,44698	0,000754
203242_s_at	PDLIM5	PDZ and LIM domain 5	−2,43851	0,001699
203868_s_at	VCAM1	Vascular cell adhesion molecule 1	−2,43513	0,000761
220206_at	ZMYM1	Zinc finger MYM-type containing 1	−2,36362	0,008439
207616_s_at	TANK	TRAF family member associated NFKB activator	−2,34567	0,000424
218303_x_at	KRCC1	Lysine-rich coiled-coil 1	−2,34567	0,003187
218490_s_at	ZNF302	Zinc finger protein 302	−2,32785	0,001816
206876_at	SIM1	Single-minded family bHLH transcription factor 1	−2,32624	0,001681
219128_at	C2orf42	Chromosome 2 open reading frame 42	−2,28628	0,002926
212861_at	MFSD5	Major facilitator superfamily domain containing 5	−2,27048	0,000823
218653_at	SLC25A15	Solute carrier family 25 member 15	−2,25636	0,000562
206943_at	TGFBR1	Transforming growth factor beta receptor I	−2,24856	0,025349
201995_at	EXT1	Exostosin glycosyltransferase 1	−2,247	0,000421
221430_s_at	RNF146	Ring finger protein 146	−2,23457	0,001084
212286_at	ANKRD12	Ankyrin repeat domain 12	−2,2253	0,00029
219544_at	BORA	Bora, aurora kinase A activator	−2,21914	0,000333
210455_at	R3HCC1L	R3H domain and coiled-coil containing 1 like	−2,2176	0,0039
219459_at	POLR3B	Polymerase (RNA) III subunit B	−2,2176	0,000832
219078_at	GPATCH2	G-patch domain containing 2	−2,19923	0,000723
204547_at	RAB40B	RAB40B, member RAS oncogene family	−2,17648	0,001741
209760_at	KIAA0922	KIAA0922	−2,17347	0,001048
218791_s_at	KATNBL1	Katanin regulatory subunit B1 like 1	−2,17347	0,001187
205173_x_at	CD58	CD58 molecule	−2,17196	0,00022
204352_at	TRAF5	TNF receptor associated factor 5	−2,16895	0,002659
212441_at	KIAA0232	KIAA0232	−2,16595	0,006084
204236_at	FLI1	Fli-1 proto-oncogene, ETS transcription factor	−2,15397	0,005141
203072_at	MYO1E	Myosin IE	−2,15248	0,000154
219904_at	ZSCAN5A	Zinc finger and SCAN domain containing 5A	−2,14801	0,00144
219133_at	OXSM	3-Oxoacyl-ACP synthase, mitochondrial	−2,12285	0,002424
205798_at	IL7R	Interleukin 7 receptor	−2,11257	0,00506
205476_at	CCL20	C-C motif chemokine ligand 20	4,613942	9,50*E* − 05
213497_at	ABTB2	Ankyrin repeat and BTB domain containing 2	4,623547	1,40*E* − 05
219179_at	DACT1	Dishevelled-binding antagonist of beta-catenin 1	4,642816	9,00*E* − 06
219228_at	ZNF331	Zinc finger protein 331	4,723971	6,00*E* − 06
213139_at	SNAI2	Snail family zinc finger 2	4,76673	1,40*E* − 05
218177_at	CHMP1B	Charged multivesicular body protein 1B	4,806544	1,00*E* − 05
203304_at	BAMBI	BMP and activin membrane-bound inhibitor	4,826576	3,00*E* − 06
201631_s_at	IER3	Immediate early response 3	4,833271	3,00*E* − 06
218559_s_at	MAFB	v-maf avian musculoaponeurotic fibrosarcoma oncogene homolog B	4,870264	0,000468
220266_s_at	KLF4	Kruppel-like factor 4 (gut)	4,890561	0,00022
209211_at	KLF5	Kruppel-like factor 5 (intestinal)	4,924578	0,002036
209681_at	SLC19A2	Solute carrier family 19 member 2	4,927992	5,90*E* − 05
205266_at	LIF	Leukemia inhibitory factor	4,955395	2,20*E* − 05
204790_at	SMAD7	SMAD family member 7	5,073566	0,000283
221667_s_at	HSPB8	Heat shock protein family B (small) member 8	5,422657	2,90*E* − 05
212665_at	TIPARP	TCDD-inducible poly(ADP-ribose) polymerase	5,525098	1,00*E* − 05
202935_s_at	SOX9	SRY-box 9	5,971114	3,30*E* − 05
202023_at	EFNA1	Ephrin-A1	6,164569	3,30*E* − 05
202393_s_at	KLF10	Kruppel-like factor 10	6,194552	0,000195
213146_at	KDM6B	Lysine demethylase 6B	6,203146	1,90*E* − 05
205193_at	MAFF	v-maf avian musculoaponeurotic fibrosarcoma oncogene homolog F	6,2941	2,00*E* − 06
209457_at	DUSP5	Dual specificity phosphatase 5	6,639157	1,30*E* − 05
206029_at	ANKRD1	Ankyrin repeat domain 1	6,65759	0,008591
209283_at	CRYAB	Crystallin alpha B	6,703897	0,000118
201693_s_at	EGR1	Early growth response 1	7,056731	4,10*E* − 05
212099_at	RHOB	ras homolog family member B	7,300524	0,000406
219682_s_at	TBX3	T-box 3	7,722136	5,80*E* − 05
201473_at	JUNB	jun B proto-oncogene	8,322402	7,00*E* − 06
200664_s_at	DNAJB1	DnaJ heat shock protein family (Hsp40) member B1	8,586082	2,00*E* − 05
205828_at	MMP3	Matrix metallopeptidase 3	8,711976	1,90*E* − 05
201169_s_at	BHLHE40	Basic helix-loop-helix family member e40	8,870405	0,00011
203665_at	HMOX1	Heme oxygenase 1	9,32433	0,000544
202643_s_at	TNFAIP3	TNF alpha induced protein 3	9,573192	2,50*E* − 05
205207_at	IL6	Interleukin 6	10,18236	3,00*E* − 06
202388_at	RGS2	Regulator of G-protein signaling 2	10,25318	1,40*E* − 05
204472_at	GEM	GTP binding protein overexpressed in skeletal muscle	10,8003	1,00*E* − 06
202149_at	NEDD9	Neural precursor cell expressed, developmentally down-regulated 9	11,06553	2,50*E* − 05
219480_at	SNAI1	Snail family zinc finger 1	11,70457	2,00*E* − 06
218839_at	HEY1	hes related family bHLH transcription factor with YRPW motif 1	12,07541	6,00*E* − 06
206115_at	EGR3	Early growth response 3	14,19194	1,20*E* − 05
204470_at	CXCL1	C-X-C motif chemokine ligand 1	17,61827	2,00*E* − 06
204621_s_at	NR4A2	Nuclear receptor subfamily 4 group A member 2	18,77837	0
209774_x_at	CXCL2	C-X-C motif chemokine ligand 2	19,02731	9,00*E* − 06
202859_x_at	CXCL8	C-X-C motif chemokine ligand 8	19,89039	1,00*E* − 06
202340_x_at	NR4A1	Nuclear receptor subfamily 4 group A member 1	20,74943	1,00*E* − 06
209189_at	FOS	FBJ murine osteosarcoma viral oncogene homolog	23,36051	1,00*E* − 06
202672_s_at	ATF3	Activating transcription factor 3	24,18432	0
202768_at	FOSB	FBJ murine osteosarcoma viral oncogene homolog B	32,92245	0
207978_s_at	NR4A3	Nuclear receptor subfamily 4 group A member 3	43,80428	1,00*E* − 06
117_at	HSPA6	Heat shock protein family A (Hsp70) member 6	90,82389	0
